# Evaluation of the Effect of Consultant Characteristics on Telemedicine Diagnosis and Treatment

**DOI:** 10.1155/2011/701089

**Published:** 2011-05-04

**Authors:** Ann B. Bynum, Cathy A. Irwin

**Affiliations:** ^1^Center for Rural Health, University of Arkansas for Medical Sciences (UAMS), UAMS Mailbox No. 599A, 4021 W. 8th Street, Little Rock, AR 72204-1611, USA; ^2^Center for Distance Health, University of Arkansas for Medical Sciences (UAMS), UAMS Mailbox No. 599A, 4021 W. 8th Street, Little Rock, AR 72204-1611, USA

## Abstract

This study examined teleconsultants' specialty, practice setting, type of employment, years and training in telemedicine to evaluate the effect of these characteristics on diagnoses and treatment. A postuse survey was conducted during 1998–2003 (*n* = 454 consultations) in the University of Arkansas for Medical Sciences' Rural Hospital, Telehealth Project. There were 61 consultants who conducted the teleconsultations. The teleconsultants established a diagnosis in 121 consultations and reported a change in diagnoses in 29 consultations. The consultants established a treatment plan in 219 consultations and reported a change in the treatment plan in 100 consultations. Dermatologists were significantly more likely to establish (*P* < .01) and change (*P* = .005) the diagnosis and to establish a treatment plan (*P* = .03), when compared to all other specialties. Teleconsultants who were self-employed were significantly more likely to change the treatment plan (*P* = .012). The findings suggest that teleconsultants' characteristics can affect diagnoses and treatment in telemedicine.

## 1. Introduction

Telemedicine can have a crucial role in enhancing health care delivery for patients in underserved areas where distance or travel challenges present barriers to specialty medical care. Physician shortage, poor access to care, and rural-urban disparities for specialty medical care need to be addressed to improve the health care of persons in underserved, rural areas. 

Telemedicine provides a potential method for addressing these problems in health care [1, 2]. Quality of medical care for patients in remote areas may be improved by using telemedicine in the delivery of specialty medical care for these patients [3, 4]. Clinical outcomes in telemedicine must be assessed to determine whether this technology improves quality of care. Improvements in clinical outcomes are related to the correct diagnosis and appropriate treatment plan [5]. The quality of medical care in telemedicine depends on the quality of the patient's diagnosis and treatment [6]. 

Delays in the diagnosis and treatment of patients in rural areas may increase morbidity and mortality rates for these patients [3]. Increased access to a specialty medical care through telemedicine may alter the patient's diagnosis and treatment plan. Previous studies have concluded that changes in the patient's diagnosis and treatment plan, as a result of the telemedicine consultation, can have an impact on clinical outcomes and patients' costs for medical care [3, 4, 7]. These studies reported that changes in diagnoses and treatment plans through telemedicine consultations can avoid morbidity, costly and inappropriate treatment, patient transfers, and patients' costs for traveling to see a specialist. As such, this study hypothesized that teleconsultants' characteristics might explain changes in patients' diagnoses and treatment plans, as a result of telemedicine consultations with medical specialists [5]. Teleconsultants' characteristics include the medical specialty, practice setting, type of employment, years and training in telemedicine, and reason for using telemedicine. 

More studies are needed to determine the clinical effectiveness or ineffectiveness of telemedicine and to determine whether improvements are made in quality of care with telemedicine. Few studies have been performed to assess the effect of telemedicine on changes in patients' diagnoses and treatment plans [1, 4–6, 8–13]. Only one of the previous studies assessed the effect of teleconsultant characteristics on changes in diagnoses and treatment plans [5]. The current study differs from this previous study, which assessed an additional outcome on clinical improvement among patients receiving telemedicine consultations. Clinical improvement was based on the consultant's assessment regarding improvement in initial symptoms and/or physical signs. This previous study examined the association between changes in diagnoses and treatment therapy and clinical improvement. Building upon previous studies, the current study evaluates the effect teleconsultants' characteristics have on rural patients' diagnoses and treatment plans and how those diagnoses and treatment plans change depending on the unique characteristics of each consultant.

### 1.1. Background

Data for this study were gathered from evaluations of the University of Arkansas for Medical Sciences' (UAMS) Rural Hospital, Telehealth Project. Telemedicine in Arkansas was initiated in 1996 to provide access to medical care for a poor, underserved, rural population in the East Arkansas Delta. For this project, specialty telemedicine consultations were delivered to patients at distant sites through a telemedical network. Interactive video technology is in every hospital in the state. The telemedicine network includes rural hospitals, eight Area Health Education Centers (AHECs), and clinic sites across the state. The project objective was to increase rural residents' access to specialty medical services across their lifespan using telemedicine and interactive compressed video technology. 

This study was conducted by the UAMS Rural Hospital Program, which was established in 1991 as an outreach program of UAMS. The purpose of this program is to strengthen rural hospitals across Arkansas by sharing resources found at the state's only academic medical center. The teleconsultant sites for the UAMS Rural Hospital, Telehealth Project included three sites in an urban setting at UAMS and two sites in hospitals located in the East Arkansas Delta. 

The purpose of this study was to evaluate the effect of consultant characteristics on telemedicine diagnosis and treatment. Teleconsultants' characteristics include the medical specialty, practice setting, type of employment, years and training in telemedicine, and reason for using telemedicine. Changes in the patient's diagnosis and treatment plan as a result of the telemedicine session were assessed. Findings from the study provided evidence for the benefits of telemedicine in upgrading patients' diagnoses and treatment plans in rural Arkansas. The findings can be used to explain changes in patients' diagnoses and treatment plans as a result of telemedicine, based on the individual characteristics of these consultants.

## 2. Methods

### 2.1. Design and Sample

This study performed a post-use survey of consultants in the UAMS Rural Hospital, Telehealth Project during 1998–2006 (*n* = 1,449 consultations). The data analyses for this study are based on the 454 teleconsultations with scanned data for the outcomes on patients' diagnoses and treatment plans during 1998–2003 (Table 1). 

The sample for this study included consultations only in grant-funded sites that participated in the evaluation of the Telehealth Project during the study period. Provider-to-provider consultations (*n* = 792) and telepharmacy education study consultations (*n* = 87) in grant-funded sites were excluded because these consultations represented a different type of service. The provider-to-provider consultations did not involve patient-provider interaction and are thus outside the scope of this study. 

Changes in the patient's diagnosis and treatment plan compared the initial diagnosis and treatment plan as established by the primary care provider and the specialist's diagnosis and treatment plan delivered during teleconsultation. The primary care provider's initial diagnosis and treatment plan for the patient were assessed in a preuse telemedicine survey. Data for the study were collected from March, 1998 to June, 2006. Procedures were approved by the Human Research Advisory Committee at UAMS.

### 2.2. Setting

The target area for the Telehealth Project consisted of seven counties in the East Arkansas Delta, with a population of 142,174 residents consisting of a large percentage (40–60%) of ethnic minorities [14]. The Delta area encompasses the poorest counties in Arkansas, which are classified as both Medically Underserved Areas (MUAs) and Health Professional Shortage Areas (HPSAs). Residents in the East Arkansas Delta often travel long distances to receive medical care in other communities ranging from 30 to 150 miles away. Moreover, many residents choose to forgo specialty medical care when travel is required, due to myriad barriers, including limited access to modes of transportation. Additionally, cultural barriers exist for many poorer and less educated residents who express feelings of intimidation from the idea of traveling to urban health care settings. These rural residents may not have the psychological, physical, or financial resources to travel to urban health care settings. 

### 2.3. Procedures

Patients were referred to the project when physicians in the target counties were unable to provide services that were needed. Primary care providers from distant, health care sites in the East Arkansas Delta requested telemedicine consults through the project coordinator. The coordinator forwarded the patient history and physical assessment data to the consultant prior to the scheduled session. 

Typically, the patient would arrive at the distant, primary care site 30 minutes prior to the consultation. The project site facilitator coordinated the session at the distant site. The assistant coordinator distributed the demographic information form for completion by the consultant at the consulting site. The consultants attended ongoing training sessions regarding telehealth procedures and interactive video/diagnostic equipment used in the telemedicine sessions. 

The distant site was equipped with interactive compressed video technology transmitted over T1 lines at 768 kbit/s bandwidth. The UAMS Rural Hospital Program provided two-way interactive video services to the distant sites. The videoconferencing equipment included Polycom View Stations (Polycom, Inc., Milpitas, CA, USA), Tandberg interactive video units (Tandberg, Lysaker, Norway), and ELMO-400 Document Cameras (ELMO, Plainview, NY, USA). 

The 30-minute telemedicine consultations involved patient interviews with the consultant and physical assessments over interactive video. The primary care provider or site facilitator presented the patient to the consultant. Follow-up care and instructions for the patient were provided during the session. Patient privacy was maintained, and telemedicine protocols for each specialty were followed during all consults. These protocols included guidelines for the telemedicine consultation equipment, referral process, scheduling, appointment confirmation, required information and evaluation forms for patients and providers, work-up protocols, and procedures for each specialty. The patient assessment included inspection and examination over interactive video, using an electronic stethoscope and a camera that can be attached to an otoscope, ophthalmoscope, and a dermascope. 

The assistant coordinator distributed the postsession evaluation and session information form for completion by the consultant at the end of the consultation session. The assistant coordinator assisted the consultant with completion of the evaluation and information forms at the consulting site. After completing the session, the consultant contacted the primary care provider to review the patient data and make recommendations regarding diagnosis and treatment.

### 2.4. Instruments

Three instruments were used to collect data: (1) consultant demographic instrument, (2) session information evaluation, and (3) consultant postsession evaluation. The 15-item consultant demographic instrument assessed characteristics of the telemedicine consultants, including telemedicine practice and training, patients referred for telemedicine consultations, and reason for using telemedicine. The 21-item session information evaluation assessed characteristics of the telehealth consultations including the clinic site, patient status, staffing, technology, and equipment. 

The 9-item, consultant postsession evaluation evaluated the consultant's established diagnosis and change in the patient's diagnosis as a result of the telemedicine session; the consultant's established treatment plan and change in the patient's treatment plan as a result of the session. The postsession evaluation instrument was developed from items that were included in previous telehealth program evaluation instruments [15]. The response scale for the two items regarding the established diagnosis included a yes, no, and not applicable responses, and the established treatment plan item allowed a yes or no response. The two items evaluating change in the patient's diagnosis and treatment plan had a response scale with yes, no, not applicable, not aware of a prior diagnosis or treatment plan, and no prior diagnosis or treatment plan.

### 2.5. Data Analysis

Data were analyzed using the Statistical Package for the Social Sciences (SPSS), Version 14. Statistical procedures included the logistic regression and chi-square test of independence. The independent variables for teleconsultants' specialty, practice setting, and reason for using telemedicine were entered into the logistic regression analysis. Separate logistic regression analyses were performed for each of these independent variables. The chi-square test of independence was used to evaluate the effect of consultants' type of employment, years and training in telemedicine on patients' diagnoses and treatment plans. The *P* value of <.05 was used as the level of significance for all statistical procedures.

## 3. Results

### 3.1. Characteristics of the Telemedicine Consultants

Results for characteristics of the teleconsultants are based on the 1,114 consultations with scanned data for responses on the consultant demographic instrument during 1998–2005 (Table 1). There were 61 teleconsultants who conducted the consultations and responded to the survey. Forty-four consultants conducted multiple consultations, ranging from two to 464 consultations. There were 33 cancelled or no-show appointments for consultations in sites that participated in this survey during the study period. The sample included five consultant sites and 16 distant, primary-care sites. 

The majority of the responding teleconsultants had received training in telemedicine: consultants in 712 (68%) teleconsultations reported that they had received training, and in 330 (32%) teleconsultations consultants reported that they had not received training, and there were 72 nonrespondents. The main reason for consultants agreeing to use the telemedicine system was their desire to improve patients' access to care: consultants in 974 (93%) teleconsultations reported that they agreed or strongly agreed with this item; in 76 (7%) teleconsultations consultants reported that they strongly disagreed; there were 64 nonrespondents. Most of the consultants had an academic medical center practice, were employed by a health care facility, and began practicing telemedicine within this system between 1996 and 1999 (Table 2). The most frequently used specialties for consultations were in obstetrics/gynecology, genetic counseling, psychiatry and psychology, nutrition and dietetics, dermatology, primary care, and pharmacy.

### 3.2. Characteristics of the Telemedicine Consultations

Results for characteristics of the teleconsultations are based on the 498 consultations with scanned data for responses on the session information evaluation instrument and on the 454 consultations with scanned data for responses on the consultant postsession evaluation instrument during 1998–2003 (Table 1). 

Most of the patients in the telemedicine consultations were outpatients: there were 474 (97%) outpatients, 1 (0.2%) inpatient, 1 (0.2%) prisoner, 11 (2%) of other status, and 11 nonrespondents. Patients were referred for further care in 236 (61%) consultations, and patients were referred to the consulting specialist who participated in the telemedicine session in 178 (53%) consultations (Table 3). Referral care was provided by telemedicine in 178 (52%) consultations. There were no reported problems with the telemedicine equipment or transmission in 442 (89%) consultations. The consultants reported problems with the equipment and transmission in a low percentage of consultations, consisting of audio and video problems; problems with the peripheral equipment (electronic stethoscope, otoscope, ophthalmoscope, and dermascope); problems with software, human error in operating equipment and other problems (Table 3).

### 3.3. Changes in the Patient's Diagnosis and Treatment Plan

The findings for patients' diagnoses and treatment plans are based on the 454 teleconsultations with scanned data for these outcomes during 1998–2003 (Table 1). Results indicated that the telemedicine consultants established a diagnosis in 121 consultations (Table 4). This was 27% of the 441 responses. Of the 102 respondents for cases where there was a prior diagnosis and a change was applicable, 29 (28%) consultants reported a change in the patient's diagnosis. The consultants established a patient treatment plan in 219 consultations. This was 52% of the 422 respondents. Of the 166 respondents for cases where there was a prior treatment plan and a change was applicable, 100 (60%) consultants reported a change in the treatment plan.

### 3.4. Effect of Consultant Characteristics on Telemedicine Diagnosis

Results from the logistic regression analysis indicated that teleconsultants who practice dermatology (*n* = 66, 71%) were significantly more likely to establish a diagnosis, when compared to all other specialties (*n* = 55, 16%) (odds ratio, OR = 13.92, 95% CI = 7.55–25.65, *P* < .01). In addition, teleconsultants who practiced in an academic medical center (*n* = 109, 31%), outpatient clinic (*n* = 27, 36%), and private office (*n* = 27, 36%) were significantly more likely to establish a diagnosis (OR = 5.72–6.87, 95% CI = 1.21–29.59, *P* = .029 to *P* < .01). Teleconsultants practicing dermatology (*n* = 15, 16%) were significantly more likely to change the diagnosis, when compared to all other specialties (*n* = 14, 4%) (OR = 4.56, 95% CI = 1.59–13.04, *P* = .005).

### 3.5. Effect of Consultant Characteristics on Telemedicine Treatment

Findings from the logistic regression analysis indicated that dermatologists delivering teleconsultations (*n* = 81, 89%) were significantly more likely to establish a treatment plan, when compared to all other specialties (*n* = 138, 42%) (OR = 2.64, 95% CI = 1.10–6.36, *P* = .03). Additionally, teleconsultants who practiced in an outpatient clinic (*n* = 40, 56%) and those who used this telemedicine system to increase their income (*n* = 10, 67%) were significantly more likely to establish a treatment plan (OR = 3.17, 8.66, resp.; 95% CI = 1.20–39.16; *P* = .02, .005, resp.). Results from the chi-square test of independence indicated that teleconsultants who were self-employed (*n* = 16, 89%) were significantly more likely to change the treatment plan (chi-square = 8.92, *df* = 2, *P* = .012).

## 4. Discussion

### 4.1. Conclusions

These results demonstrate that telemedicine had an effect on changing the patients' diagnoses and treatment plans during consultations in the UAMS Rural Hospital, Telehealth Project. Although changes in the patients' diagnoses and treatment plans are not direct measures of quality, these results imply that the patients' diagnoses and treatment plans were upgraded as a result of the teleconsultations for a rural population in Arkansas. The accuracy of the consultants' diagnoses and treatment plans could have an effect on the quality of the patient's medical management during the telemedicine session. Future studies would be needed to assess the accuracy of the consultants' diagnoses and treatment plans in the Telehealth Project. In addition, further research is needed to assess the association between changes in the patient's diagnosis and treatment plan and the likelihood of clinical improvement. 

The results suggest that teleconsultants' characteristics can affect diagnosis and treatment through telemedicine. The consultant's specialty might explain the results for changes in the patients' diagnoses and treatment plans. The most frequently used specialties for consultations were in obstetrics/gynecology, genetic counseling, psychiatry and psychology, nutrition and dietetics, dermatology, primary care, and pharmacy. Telemedicine consultations for these specialties may require changes in the patient's diagnosis and treatment plan more often than for other specialties. Variability in the consultants' practice and management of patients' health problems might also affect the changes found in the patients' diagnoses and treatment plans as a result of the telemedicine session. Changes in the patient's diagnosis and treatment plan were not applicable in some of the teleconsultations that focused on health counseling for nutrition, medications, and other health education needs. 

The teleconsultants' motivation for using telemedicine may have affected the results of this study. Consultants used the telemedicine system to improve patients' access to care, and consultants who used the telemedicine system to increase their income were more likely to establish a treatment plan. These motivational factors may have affected the results for changes in the patients' diagnoses and treatment plans. 

The telemedicine consultations were provided for patients in the East Arkansas Delta who have limited access to medical specialists. The patients' barriers to care might explain the changes that occurred in the patients' diagnoses and treatment plans. In addition, the majority of the consultations had no problems with the equipment or transmission. The lack of problems with the diagnostic equipment and transmission used in the consultations could have an impact on the findings that indicated changes in the patient's diagnosis and treatment plan as a result of the telemedicine session. 

Few studies have been performed to assess the effect of telemedicine on changes in the patient's diagnosis and treatment plan [1, 4, 5, 8–13]. Results of this study are consistent with previous studies that found changes in the patients' diagnoses and treatment plans as a result of telemedicine consultations. Only one of the previous studies assessed the effect of teleconsultant characteristics on changes in the diagnosis and treatment plan [5]. This study conducted a retrospective review of 223 individual telemedicine medical records and found that teleconsultations resulted in changes in diagnoses in 48% of the patients, changes in the treatment plan in 82% of the cases, and clinical improvement in 60% of the patients. Changes in the diagnosis and treatment plan were associated with clinical improvement. Dermatology had the highest percentage of changes in the diagnosis, and psychiatric consultations had the highest percentage of changes in the treatment plan. Clinical improvement also varied by consultant specialty. 

Findings from a prospective study indicated that teleradiology (*n* = 685 cases) changed the treatment in 22 cases out of the 42 cases with a false interpretation [4]. In almost two-thirds of the cases the teleradiology consultation helped with the diagnosis, and a new diagnosis was made in 4% of cases. An 8-month prospective study compared orthopedic surgery outpatient clinics with telemedicine to those clinics without telemedicine (*n* = 419 patients) [8]. The findings demonstrated that the diagnoses were revised to a similar degree after orthopedic surgery teleconsultations (12%) and after face-to-face clinic visits (16%) when compared with the diagnoses established by the general practitioners. Results from an experimental study (*n* = 108 patients with 123 acute fractures) indicated that additional information provided by viewing electronically transmitted images of radiographs changed the acute management or the ultimate management in 21% of the fractures [16]. 

An evaluation of a pulmonary telemedicine clinic (*n* = 314 patients) demonstrated that teleconsultations resulted in changing the treatment plan for 41% of patients [1]. Findings from a post-use survey of a telehealth project (*n* = 412 consultations) indicated that 27% of the consultants reported a change in the diagnosis and 67% of the consultants reported a change in the treatment plan [9]. An additional investigation evaluated pediatric, echocardiography studies (*n* = 769) conducted by telemedicine and found a change in the diagnosis in 10 videotaped studies that were attributed to lack of diagnostic clarity, when compared with on-site studies [10]. The diagnostic accuracy of telepathology consultations on 1,255 static image-based cases was evaluated over a period of 5 years [11]. Results indicated that there was a clinically significant concordance rate of 97.3% between telepathology and final diagnosis and an absolute concordance rate of 73.7% was achieved. 

Findings from a pre-post study of a telepsychiatry counseling service for youths (*n* = 190) in a juvenile detention facility indicated that treatment, behavioral goals for each adolescent increased in the first and second year of this project [12]. An additional study conducted a retrospective medical record review of patients (*n* = 99) referred to a pediatric telemedicine weight management clinic [13]. The teleconsultations resulted in changes to diagnoses in 78% of patients; 81% demonstrated improvement in clinical outcomes, and changes to treatment were associated with improvement in weight status. 

### 4.2. Implications

This study provided evidence that telemedicine changes the patient's diagnosis and treatment plan for an underserved, rural population in the East Arkansas Delta. Telemedicine promoted access to medical specialists in the patients' local community. The changes that occurred in the patient's diagnosis and treatment as a result of the telemedicine session may have avoided delays in the appropriate diagnosis and treatment for these patients. Telemedicine facilitated more confident local management of patients in this rural area of Arkansas. The telemedicine consultations provided educational and training opportunities for the referring primary care provider and other health professionals involved in the consultation. The consultants gave advice for these health professionals on aspects of the patients' diagnoses and treatment plans. The results can be used to improve the telemedicine procedures in the Telehealth Project. 

Results from this study present clinical and political implications for telemedicine in rural, underserved areas. The findings reinforce the value of using dermatologists to deliver teleconsultations because these consultants were more likely to establish and change the patients' diagnoses and to establish the treatment plans. Additional results support the use of teleconsultants who practice in an academic medical center, outpatient clinic, and private office and those who are self-employed and use the telemedicine system to increase their income. These teleconsultants were more likely to establish a diagnosis and treatment plan. The results may be used to provide evidence for the reimbursement and allocation of funds and resources for these consultants in delivering telemedicine for a rural, underserved population in the Arkansas Delta. 

The consultants in this project were paid for each teleconsultation. Patient billing for the teleconsultation was made as for any other clinic billing. The patients' insurance company was billed for each teleconsultation. The grant funding provided payment for teleconsultations for patients without insurance. 

Additional implications involve the use of teleconsultants who have experience and training in telemedicine. Most of the consultants in this project began practicing telemedicine within the system between 1996 and 1999 and had received training in telemedicine. Telemedicine consultation with an experienced and trained specialist may have an impact on the consultant's confidence in establishing and changing the patient's diagnosis and treatment plan. Training in telemedicine for less-experienced consultants is recommended for improving their confidence in diagnosis and treatment during teleconsultations. 

Limitations of the study design restrict the generalizability of the results. The study design also limited the ability to determine the diagnostic and therapeutic effectiveness of the telemedicine procedures used in the Telehealth Project. Nonrespondents for study variables were also limitations. 

There is no risk for bias due to nonrespondents in this study. The number of nonrespondents for variables on patients' diagnoses and treatment plans ranged from 13 to 171. There was nothing selective about the sample of respondents. The data analyses for this study are based on the 454 teleconsultations with scanned data for the outcomes on patients' diagnoses and treatment plans during 1998–2003. Data on outcomes for patients' diagnoses and treatment plans, for years 2004–2006, were not scanned in the data base. 

Implications for future research include methods for decreasing the nonrespondents for study variables. Program methods are suggested for future studies that involve instructions for consultants regarding completion of all instrument items for evaluation of the teleconsultations and teleconsultants. Additional recommendations include ongoing training for site facilitators on these methods of instrument distribution. Future studies are recommended that use an experimental design to compare telemedicine consultations and face-to-face medical care on the patient's diagnosis and treatment plan in remote, rural communities and among different ethnic groups. Further research is needed to assess the impact of telemedicine on improvement in clinical outcomes and to examine the association between changes in the patient's diagnosis and treatment plan and the likelihood of clinical improvement.

## Figures and Tables

**Table 1:** Time periods for data collection in the UAMS Rural Hospital, Telehealth Project.

Variable	Time period
Patients' Diagnoses and Treatment Plans	1998–2003
Characteristics of the Teleconsultants	1998–2005
Characteristics of the Teleconsultations	1998–2003
Patients' Diagnoses and Treatment Plans—Data were not scanned in the data base	2004–2006
Characteristics of the Teleconsultants—Data were not scanned in the data base	2006
Characteristics of the Teleconsultations—Data were not scanned in the data base	2004–2006

**Table 2:** Characteristics of the Teleconsultants in the Teleconsultations (*n* = 1,114).

Variable	*n*	Percentage (%)
*Primary practice setting		
Academic medical center	992	89
Hospital	406	36
Outpatient clinic	423	38
Private office	374	34
Community health clinic	364	33

Consultant—years in practice		
1–9 years	575	56
10–45 years	458	44
No response	81	

Type of employment		
Health care facility	991	95
Provider organization	24	2
Self-employed, other	33	3
No response	66	

Date telemedicine practice began within this system		
1996–1999	357	51
2000–2003	336	49
No response	421	

Consultant specialty		
Obstetrics/Gynecology	455	31
Genetic Counseling	349	24
Psychiatry/psychology	210	15
Nutrition and dietetics	113	8
Dermatology	109	8
Primary Care	59	4
Pharmacy	56	4
Other 17 specialties	98	7

Consultant discipline		
MD	483	41
MSc	300	25
PhD	203	17
PhD, registered dietician	86	7
PharmD	56	5
Health related professions	32	3
Registered nurse	19	2
Social worker	2	.10
Physical therapist	1	.08
No response	267	

Number of patients seen using telemedicine		
≤30 patients	253	23
>30 patients	834	75
First consult	27	2

*Note. Consultants could choose multiple answers for this item.

**Table 3:** Characteristics of the Teleconsultations (*n* = 454).

Variable	*n*	Percentage (%)
Patient referred for further care		
Yes	236	61
No	149	39
No response	69	

Patient referred to		
Hospital emergency room	13	4
Participating/referring primary provider	35	11
Another primary provider	1	.30
Another consulting specialist	12	4
Consulting specialist in the telemedicine session	178	53
Other	17	5
Not applicable	77	23
No response	121	

Referral care		
In-person in another community	34	10
In-person in the patient's community	40	12
Via telemedicine	178	52
Not applicable	76	22
Don't know	4	1
Other	8	2
No response	114	

*Problems with equipment or transmission		
No problems	442	89
Problems with audio	12	2
Problems with video	20	4
Problems with peripheral equipment	7	1
Problems with software	1	.20
Human error in operating equipment	3	.60
Other problems	8	2

Effect of problems with equipment or transmission during the consultation		
Minimal effect	99	24
Moderate effect, delayed session	29	7
Moderate effect, session difficult	6	1
Significant effect, very disruptive	4	.90
Impossible to complete session	1	.20
Not applicable	280	67
No response	79	

*Note. Consultants could choose multiple answers for this item.

**Table 4:** Changes in the Patient's Diagnosis and Treatment Plan as a Result of the Telemedicine Session (*n* = 454 teleconsultations).

Variable	*n*	Percentage (%)
Established diagnosis as part of this session		
Yes	121	27
No	320	73
No response	13	

Change in the patient's diagnosis		
Yes	29	10
No	73	26
Not aware of a prior diagnosis	25	9
There was no prior diagnosis	17	6
Not applicable	139	49
No response	171	

Established treatment plan as a result of this session		
Yes	219	52
No	203	48
No response	32	

Change in the patient's treatment plan		
Yes	100	29
No	66	19
Not aware of a prior treatment plan	40	12
There was no prior treatment plan	27	8
Not applicable	114	33
No response	107	
